# Research “recover from illness defense complex” helper T cell immune mechanisms based on the “Fuxie” theory clearing away heat evil thoroughly nourishing kidney treatment of recurrent blood-heat syndrome Psoriasis

**DOI:** 10.1097/MD.0000000000020161

**Published:** 2020-05-15

**Authors:** Mao Li, Xia Hu, Ping-Sheng Hao

**Affiliations:** Hospital of Chengdu University of traditional Chinese Medicine, Chengdu, China.

**Keywords:** cellular immune mechanism, Chinese herbal medicine, Jia Wei Liang Xue Xiao Feng San, psoriasis, recurrent

## Abstract

**Introduction:**

Psoriasis vulgaris (PV) is a chronic, painful, disfiguring, and disabling dermatological disease, which affects the physical and mental health of patients and impacts their quality of life. Current conventional systemic therapies can be costly, present risks of side effects, have limited efficacy and commonly recur following treatment cessation. Some Chinese herbal medicine therapies have shown therapeutic benefits for psoriasis vulgaris, including relieving symptoms and improving quality of life, and a potential of reducing relapse rate. However, explicit evidence has not yet been obtained.

**Methods and analysis:**

This is a pilot randomized controlled trial with the objective of investigating the effect of Jia Wei Liang Xue Xiao Feng San granules on relapse rate of recurrent PV and the correlation between Psoriasis area severity index (PASI) and key psoriasis-related cytokine changes and the number of cells. A total of 102 participants were recruited for this study, including 72 patients with recurrent PV, 15 healthy volunteers and 15 patients with psoriasis vulgaris who have recovered for more than 1 year. A total of 72 patients, with recurrent PV, will be randomized (1:1) to receive the oral Chinese herbal medicine Jia Wei Liang Xue Xiao Feng San or the oral Acitretin Capsule treatments for a period of 8 weeks. After this period, participants whose PASI scores improvement reached more than 75%, will undergo a 52-week follow-up phase.

The primary outcome measures are as follows:

(i) PASI change (%) from baseline to the end of treatment phase;
(ii) key psoriasis-related cytokine changes (e.g., IL17F, IL22 and IFN-γ) and the number and distribution of cells (e.g., Th1, Th2, Th17, Treg, Th22, and Th9); and
(iii) relapse rates at the end of the follow-up phase.

The secondary study outcomes will include:

(i) The dermatology life quality index (DQOLS);
(ii) the score of pruritus degree; and
(iii) safety.

This trial may provide a novel regimen for recurrent PV patients if the granules decrease recurrence rate without further adverse effects.

**Ethics and dissemination:**

The ethics approval was provided by the Sichuan Traditional Chinese medicine regional ethics review committee. The ethics approval number is 2018KL-055. The design and the results of the study will be disseminated through peer-reviewed publications and conference presentations.

**Trial registration number:**

Chinese Clinical Trial Registry (ChiCTR1900022766).

## 1 Introduction

Psoriasis is an immune-abnormal, chronic, proliferative skin disease,^[1]^ characterized by a well-defined and raised red patches (plaques) with adherent thick silvery scales. The cause of this disease is still not well known. This disease can involve the skin of the whole body that can be accompanied by nail and joint lesions, which relapse rates in the Chinese population result in a greater severity, affecting the physical and mental health of patients and impacting their quality of life. Due to the lack of clinically specific remedy, this disease has always been one of the most important diseases in the field of dermatology.^[2]^ The recurrence of psoriasis is a difficult problem for dermatologists and patients with psoriasis. Therefore, some scholars have purposed that the treatment is to control the development of psoriasis at an early stage to reduce the area involved, achieve and maintain long-term remission, and minimize side effects to improve patients’ quality of life.^[3]^

At present, routine therapies typically involve the application of local and systematic treatments. Few therapies were accepted by patients, especially systemic drugs. Although often effective, these are cautiously used, owing to concerns surrounding their safety profile, their long-term use and the frequent relapse after drug withdrawal. Chinese herbal medicine has been frequently used for symptoms associated with psoriasis. Clinical practices have proved that TCM is beneficial and effective in alleviating psoriasis clinical symptoms, improving quality of life, immune function, reducing metastasis and in preventing recurrence in various diseases.^[4]^ However, a rigorously designed, randomized and controlled trial to investigate how traditional Chinese Medicine can potentially reduce recurrence rate is required.

There are very few studies that investigated psoriasis mechanism of recurrence at home and elsewhere. The Carrascosa et al^[5]^ study found that the decrease in the number of CD4 cells was statistically significant with a persistent psoriasis remission. Casteljns et al^[6]^ confirmed that keratin 16 is significantly expressed in the asymptomatic skin, adjacent to a recurrent lesion, that Ki-67 expression increased at the edge of skin lesions and that keratin 14 is expressed in recurrent lesions at the initial recurrence stage of psoriasis. Flisiak et al^[7]^ confirmed that plasma IL- 18 concentration was associated with the severity of psoriasis and that the combined determination of plasma IL- 18 and TGF -β1 can be used as a possible sign of psoriasis activity. Friedrich et al^[8]^ also confirmed that the treatment with the immunoregulating IL-10 can reduce the recurrence rate and prolong the interval of psoriasis recurrence. We believe that the immune mechanism of psoriasis recurrence mainly focuses on the innate immune and acquired immune systems. A large number of basic studies have shown that psoriasis is an organ-specific inflammatory disease, where T cells dominate its pathogenesis and persist its inflammatory status.^[9]^

According to the traditional Chinese medicine theory and clinical observation, the Chinese medicine compound Jia Wei Liang Xue Xiao Feng San (JWXXF) was developed by a renowned Chinese herbal medicine clinician (Professor Ai-Ru Di), and that the experimental results confirmed that^[10,11]^ JWXXF can have a positive effect in psoriasis treatment by inhibiting KC keratinization, preventing inflammation, inhibiting PCNA expression and regulating TNF-1. Therefore, a rigorously designed, randomized and controlled trial to investigate the possibility and degree of JWLXXF granules to reduce recurrence rate and to provide immunological basis for JWLXXF granules application in psoriasis recurrence treatment is required.

## 2 Methods/design

**Figure 1:**
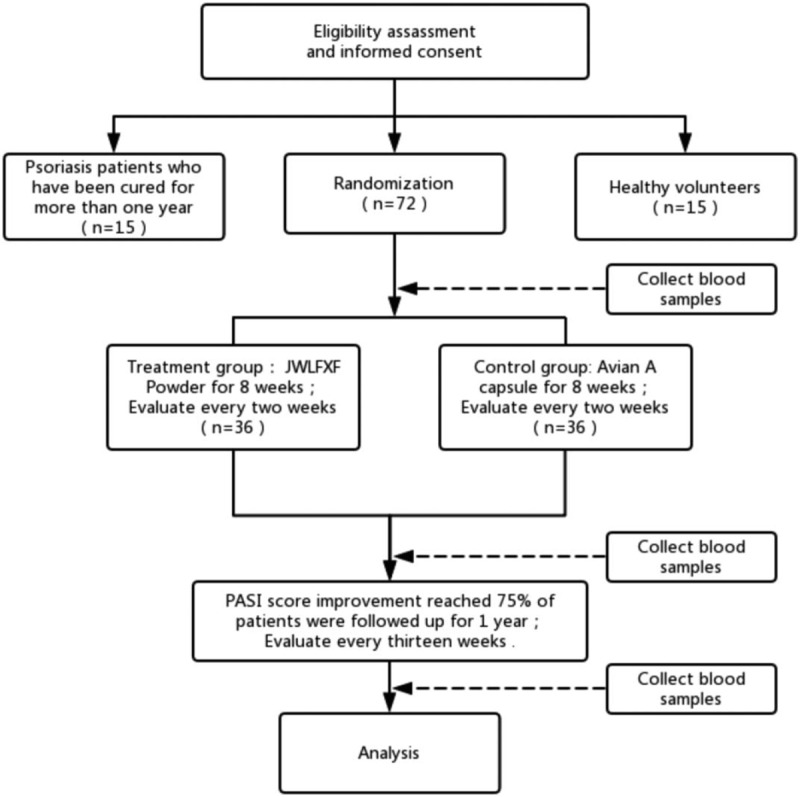
Test flow chart.

This is a pilot randomized controlled trial for 3 years. In this study, a total of 102 participants will be enrolled, including 72 patients with recurrent PV, 15 healthy volunteers and 15 patients with psoriasis vulgaris who have recovered for more than 1 year. The entire trial consists of an initial assessment, a one-week screening phase (week 1), an 8-week observation phase (week 1 to week 8) and a 52-week follow-up phase (week 9 to week 60) (Fig. 1).

Potential participants will be invited to an initial assessment (first visit) consisting of an inclusion and exclusion criteria screening. If eligible, participants will be asked for informed consent and will undergo assessment of Psoriasis area severity index (PASI), dermatology life quality index (DQOLS), and score of Pruritus Degree by PGA, lesion photos and vital signs, full blood count, kidney and liver function blood tests, electrocardiogram, stool examination and urine (routine chemical examinations).

A total of 72 patients with recurrent PV will be randomized (in a 1:1 ratio) to receive oral Chinese herbal medicine JWLXXF or oral acitretin for 8 weeks. During the observation phase, psoriasis symptom measures will be self-reported by participants and recorded in a daily or weekly diary. Face-to-face assessments with blinded assessors will be scheduled at weeks 2, 4, 6, and 8 (Fifth visit), with further data collected, including assessment of PASI, DQOLS, and score of Pruritus degree by PGA, lesion photos, drug adherence, vital signs, adverse events and cause analysis of shedding (Table 1). The conversation will be made to 72 patients, at the time of medical treatment, to check adherence to the dosage guidelines and to monitor adverse events. Routine chemical examinations, laboratory assessments of efficacy and safety evaluation will also be scheduled at the end of the 8-week period.

**Table 1:**
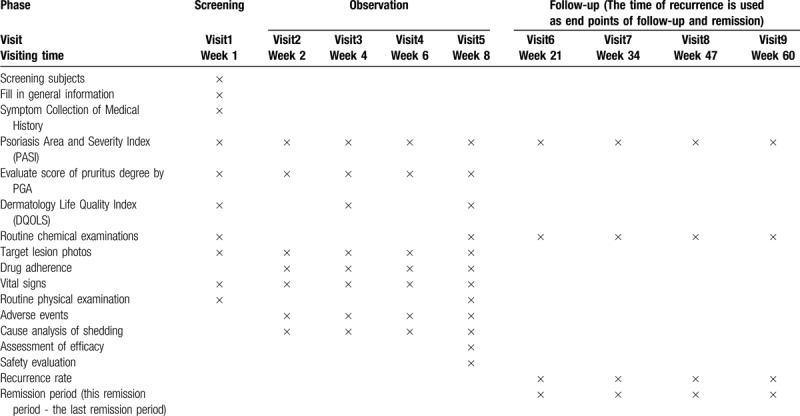
Evaluation measures and time points.

The 72 patients with recurrent PV, whose PASI scores improvement are more than 75%, will undergo a 52-week follow-up phase (week 9 to week 60), with further data collection, including assessment of PASI, routine chemical examinations, recurrence rate, remission period (This remission period - the last remission period) (Table 1).

For the 52-week follow-up phase, participants will continue to record symptoms in their daily or weekly diaries. At week 60, participants will attend an end of follow-up assessment (9th visit) which will mark the end of the trial.

## 3 Setting and participants

There are 3 types of subjects in this study: 72 patients with recurrent PV, 15 healthy volunteers and 15 patients with psoriasis vulgaris, who have recovered for more than 1 year.

The recruitment will be performed through posters in local newspapers and hospitals and participants might also be invited to contact trial coordinators via their treating physicians (general practitioners, dermatologists, or immunologists). Research assistants will introduce and discuss the trial to potential subjects. They will be given a consent form and separate information sheets, including information on the main aspects of the trial. Patients will then be able to have an informed discussion with their family and participating consultants. The research assistants will obtain the signed consent from patients willing to participate in the trial.

### 3.1 Eligibility criteria

#### 3.1.1 Inclusion criteria

Patients will be included if they satisfy the following criteria:

(1) comply with the diagnostic criteria for psoriasis vulgaris as indicated in dermatovenerology 8th Edition;
(2) comply with the diagnostic criteria for blood-heat syndrome as indicated in the Guidelines for the Clinical Research of Chinese Medicine New Drugs and New Dermatology of Traditional Chinese Medicine;
(3) must be aged between 18 and 70 years;
(4) An informed consent is provided; and
(5) recurrent psoriasis patients must belong to the types of recurrence IV, V and VI^[12,13]^: Type IV (frequently limited type): The rash is extensive or limited at the first onset, and rashes occur every year afterward, but the amount of rash is small and is limited to certain parts of the body; Type V (frequently sparsely sparse): Psoriasis often recurs, with a wide range of rashes at the onset, but with a sparse density; Type VI (frequently issued): Psoriasis occurs every year, and the rash is widely distributed and numerous.

#### 3.1.2 Exclusion criteria

People with any of the following will be excluded from participation:

(1) psoriasis with other skin diseases;
(2) those with a known cancer, infection, electrolyte imbalance, acid-base disturbance or calcium metabolic disorder, known disorders of calcium metabolism (high blood calcium levels);
(3) psychotic patients (self-assessment anxiety scale (SAS) scores > 50 or self-assessment depression scale scores > 53, or other psychiatric scores), hyperlipidemia, high vitamin A, pregnant and lactating, or plan to become pregnant within a year;
(4) types of psoriatic arthritis or pustular psoriasis or erythroderma psoriaticum;
(5) taking systemic drugs or phototherapy for psoriasis within the 4 weeks prior to screening and taking topical drug treatment for psoriasis within the 2 weeks prior to screening.

#### 3.1.3 Elimination criteria

People with any of the following will be excluded from participation:

allergic to drugs used in the study;

cannot take medication and reviewers as required;

incomplete recording or automatic suspension of treatment;

during the experiment, the patient had a major negative event, such as illness or major mental stimulation.

#### 3.1.4 Inclusion criteria of healthy volunteers

Healthy volunteers’ inclusion criteria:

(1) aged between 18 and 65 years;
(2) no family history of psoriasis;
(3) people who did not use corticosteroids or immune agents within a month of the trial;
(4) people without serious systemic diseases, tumors, immunodeficiency, or mental disorders.

### 3.2 Interventions

The intervention group will receive individually packaged doses (n = 6 g) of JWLXXF, with each dose to be dissolved in warm water and orally consumed three times a day for 8 weeks. The control group will take 10 mg of Acitretin once every day for 8 weeks. The oral Chinese herbal medicine granule JWLXXF will be produced by the Department of Pharmacy, Sichuan Hospital of traditional Chinese Medicine, a manufacturer holding a Good Manufacturing Practice certificate. The Acitretin will be provided by the Chongqing Huabang Group, number H20010126.

### 3.3 Concomitant treatment

According to the doctor's advice, white Vaseline can be used as a basic concomitant treatment to relieve dry skin and without taking other topical drugs during treatment.

### 3.4 Randomization

Unpredictable randomized sequences were generated by computer and sealed in opaque and sequentially encoded envelopes. After determining the eligibility of the participants, the researchers will break the envelopes in order and assigned them to the appropriate test group. The person who will generate the random sequence will not be the same person as the assigned subject, and the random assignment will not be displayed until the end of the study.

### 3.5 Outcome measures

#### 3.5.1 Primary outcomes

(1) PASI: assess the severity of the disease.
(2) Laboratory assessments: the number and distribution of Th1, Th2, Th17, Treg, Th22, and Th9 cells will be determined by ELISA. The expression of the transcription factors T-bet, GATA3, ROR gamma t, Foxp3 gene will be investigated by PCR and WB. The screening of cytokines, such as IFN-gamma, IL-4, IL-17A, IL-17F, IL-22, TGF-beta, IL-10 and IL-9 will be performed by Bio-Plex suspension chip.
(3) Recurrence rate = recurrence cases/follow-up cases ∗100%

Note:

No recurrence criteria: the same as the end of treatment and lesion limitation. Recurrence criteria: new skin lesions appearing in the patients who were clinically cured or have the original skin lesions expanding, the color turning red, the scale increasing and the PASI score increasing for ≥ 1.

(1) Mitigation period: this remission period - the last remission period.

Note:

The last remission period: the time from the last clinical cure to the recurrence. This remission: the time interval from the end of JWLXXF treatment to the next recurrence.

### 3.6 Secondary outcomes

(1) The DQOLS: the DQOLS 4-point scale contains 30 questions, involving psychological activities, such as embarrassment, despair, stress, pain (“very” to “no”) and social activities, such as daily life, social life, sexual life (“very bad” to “normal” or “very few” to “often”) and scoring 1 to 4 points from right to left. The cumulative score ranges from 30 to 100.
(2) Score of pruritus degree: patient global assessment (PGA) “How serious is your skin pruritic now?” The patient's answer ranges from “no pruritic” to “intensively pruritic” and can be given with a score of 0 to 6.
(3) Safety Assessment: the routine chemical examinations will be performed in 72 patients, at week 1 and 8 and will involve blood, urine analysis and fecal routine analyses, electrocardiogram, kidney and liver function assessments (ALT, AST, BUN, Cr, TBIL, GLU).

### 3.7 Adverse events

(1) Ask and record the adverse events and degree of patients at each consultation after medication, and record, in details, the symptoms, occurrence, duration and disappearance times of adverse events.
(2) The correlation between adverse events and drugs is assessed according to whether they are unrelated, possibly unrelated, possibly relevant, or relevant.
(3) Incidence of adverse events = number of adverse events / total cases × 100%.
(4) After treatment, the patients’ tolerance to drugs will be evaluated according to poor, moderate, light and high praise.

If adverse events occur or there were abnormal blood test results of liver and kidney function, the subjects will be advised to discontinue all drug treatments related to the test and seek medical advice from doctors for a safe continuation of the study.

### 3.8 Sample size calculation

Due to the unknown potential primary outcome at this stage, the sample size was calculated by reference to a standardized effect size. An effect size of 0.9 SD was chosen with consideration of the drug cost and patients’ motivation to adhere to the treatment. To detect a difference of 0.9 SD with 90% power using a 5% significance level, a sample size of 30 per arm would be required. Considering 20% dropout rate, the total sample size should be adjusted to 36 for each group.

### 3.9 Data management and quality control

In this study, the data will be collected from participants through face-to-face assessment and recorded in the case report form. The original recorded data in the case report form cannot be changed at will. If it is necessary to make corrections, it shall use the correct writing format with the signature and time on the name of the person who made the change. At the end of each evaluation, all data will be added into the database by the people who did not know the task of the team nor did participate in the case collection. The researchers who will participate in the experiment will receive advance training and education, to ensure a high experimental quality. The experimental supervision will be commissioned by the Sichuan provincial Chinese medicine clinical trial organization.

### 3.10 Statistical analysis

SPSS Statistics 17.0 (IBM SPSS Inc., Armonk, NY) will be used for the statistical analysis of data, which will be carried out by independent statisticians. The Chi-square test will be used to evaluate the basic baseline characteristics and primary outcomes of the 2 groups. The Spearman rank correlation analysis of the correlation between PASI and biochemical indexes, will be used. The secondary outcomes will be evaluated by means, standard deviations and medians. The one way ANOVA test will be used to compare the mean of multiple samples, the LSD test for the homogeneous variance, and the Tamhane T2 test for the uneven variance. *P* < .05 will be considered as statistically significant.

## 4 Discussion

### 4.1 Prescription selection basis

Chinese herbal medicine has a long history in Asia and several in vivo studies, related to psoriasis, have also proved that Chinese herbal medicine has the advantages of prolonging the remission period, with lower side effects and a reduced recurrence. However, the exact mechanism of its action is still unclear and the impact on recurrence rate has not been confirmed. Our previous study showed that the JWLXXF compound was used to treat 30 cases of psoriasis vulgaris in the progressive stage of blood heat syndrome.^[14]^ After 2 courses of treatment, 22 cases were remarkably effective, 7 cases were partially effective, and 1 case was ineffective. The total significant efficiency rate was 73.33% and the total efficiency rate was 96.67%. After performing statistics, the curative effect is slightly better than that of the compound Qingdai capsule group. Modern pharmacology shows that the JWLXXF compound is mainly aimed at the pathogenesis and pathological changes of psoriasis. The main drugs have anti-inflammatory effects. Rehmannia, buffalo horn, Hedyotis diffusa and Glycyrrhiza also have immunomodulatory effects. Peony skin, Hedyotis diffusa, Forsythia suspense and Glycyrrhiza affect tumors by providing tumor growth resistance. For example, Glycyrrhiza can inhibit tumors by reducing the level of TNF-α. In addition, Bombyx mori and mulberry leaves can reduce blood sugar; Ligustrum lucidum can adjust blood lipid; and Ligustrum lucidum and Eclipta can protect the liver.^[15,16,17,18,19,20,21,22,23,24,25,26,27,28]^ Later experimental studies also showed that the mechanism of JWLXXF may be achieved by inhibiting KC keratinization, providing anti-inflammatory benefit, inhibiting PCNA and regulating TNF-α. Therefore, JWLXXF is a result of a combination of clinical experience, pharmacological and scientific research.

### 4.2 Clinical efficacy evaluation basis

For moderate and severe psoriasis, PASI score is still the “gold standard” to evaluate the severity of psoriasis.^[29]^ According to the research, patients with sustained and efficient response to PASI score have a better quality of life score.^[30]^ According to the requirements of the new drug effective standard, after three months of test, the effective standard is a 75% improvement of the PASI score, which is called PASI75.^[29]^ PASI75 combined, with the pruritus score and DQOLS, can evaluate the short-term efficacy of JWLXXF. It needs a certain time to follow up on the recurrence rate and remission period to determine the long-term effect. After 8 weeks of treatment, patients with a pai75 will be followed up and this period will start from the end of the treatment. If there is no recurrence, the follow-up period is 1 year. If there is recurrence, the recurrence of the disease is the end point of the follow-up.

According to the guiding principles for clinical research on new drugs of traditional Chinese medicine (Part III), issued by the Ministry of health in 1997 and the directions of new drugs clinical research (try out) issued by the State Food and Drug Administration in 2002, the recurrence criteria are: new skin lesions appear in the patients who are cured clinically, or the original skin lesions expand, turn red, scale increase, and PASI score increase for ≥1. No recurrence criteria: the same as that at the end of treatment, but with limited skin lesions.^[31]^

### 4.3 Test technology design basis

From the cellular, molecular, proteic and genomic perspectives, the mechanism of the JWLXXF compound in the treatment of psoriasis and its role in recurrence prevention was discussed. The target of the drug was comprehensively analyzed, so that the experiments could complement and confirm each other. ELISA, WB and PCR technologies are the basic means of scientific research and application in dermatology. The method of liquid-phase protein chip organically integrates microsphere, laser detection technology, fluid dynamics, high-speed digital signal processing system and computer operation function. Not only the detection speed is very fast, but it also provides specificity and sensitivity in immunodiagnosis and protein molecular interaction analysis that are often beyond the conventional technology.

### 4.4 Trial status

The recruitment phase began in April 2019. Thus far, 60 patients have been recruited. The estimated end date for this study is December 2019.

## Acknowledgments

The authors would like to express their gratitude to EditSprings (https://www.editsprings.com/) for the expert linguistic services provided.

## Author contributions

**Data curation:** Mao Li.

**Formal analysis:** Xia Hu.

**Funding acquisition:** Ping-sheng Hao.

**Investigation:** Mao Li.

**Methodology:** Mao Li.

**Software:** Xia Hu.

**Validation:** Xia Hu.

**Writing – original draft:** Mao Li, Xia Hu.

**Writing – review & editing:** Ping-sheng Hao.
